# “I FEEL LIKE A SOMEBODY AGAIN”: THE ETHICS OF CARE IN A SHELTER FOR OLDER ADULTS FLEEING ABUSE

**DOI:** 10.1093/geroni/igae098.1107

**Published:** 2024-12-31

**Authors:** Lena Rebecca Richardson, Sarah Canham, Rachel Weldrick, Jill Hoselton, Alison Grittner, Christine Walsh

**Affiliations:** Simon Fraser University, Vancouver, British Columbia, Canada; University of Utah, Salt Lake City, Utah, United States; McMaster University, Hamilton, Ontario, Canada; University of Calgary, Calgary, Alberta, Canada; Cape Breton University, Sydney, Nova Scotia, Canada; University of Calgary, Calgary, Alberta, Canada

## Abstract

Elder abuse is one pathway into homelessness for older adults. Given the limited research on the experience of older adults staying in homeless shelters for victims of abuse, we conducted qualitative interviews with ten clients and five providers from an elder abuse shelter in Western Canada. Drawing on a feminist ethics of care framework, we explored what supports older survivors of abuse use to cultivate a sense of belonging at the shelter. Conducting a secondary qualitative data analysis, we identified three themes: 1) trust-building between providers and clients supports successful care-receiving by clients; 2) mutual care and caregiving by clients fosters clients’ sense of belonging; and 3) a perceived lack of care disrupts trust and impedes belonging. Our findings reveal how client trust is, in part, cultivated by providers’ acts of flexible, responsive care, supported by organizational policies and practices. In addition, clients’ acts of mutual care and caregiving towards other clients, friends, family, strangers, and animals contributes to their sense of belonging. In contrast, a perceived lack of care could be a source of conflict, though such experiences can be repaired through responsive care by providers and clients. Findings highlight the importance of shelter policies and practices which conceptualize older clients fleeing abuse as part of a multidirectional web of caring relations in fostering belonging. This study has implications for shelter program design that prioritizes trust-building through flexible, responsive care by providers, and recognizes client needs for care-receiving, caregiving, and mutual care.

